# Editorial: Climate change and sports events adaptations

**DOI:** 10.3389/fspor.2025.1706627

**Published:** 2025-10-22

**Authors:** R. Cayolla, S. Trendafilova, M. Escadas, T. Daddi, J. M. Casper

**Affiliations:** ^1^ Portucalense University, Porto, Portugal; ^2^ The University of Tennessee, Knoxville, TN, United States; ^3^ University of Minho, Braga, Portugal; ^4^ Institute of Management at the Sant'Anna School of Advanced Studies, Pisa, Italy; ^5^ North Carolina State University, Raleigh, NC, United States

**Keywords:** climate change, sports events, sustainability, triple bottom line, adaptations

**Editorial on the Research Topic**
Climate change and sports events adaptations

## 1 Introduction

In the new global economy, sustainability has become a central issue both in academia and practice. Nowadays, it is no longer enough for organizations to act and demonstrate their commitment with the environment; they must also show real involvement, a desire to improve practices, and the ability to communicate their efforts in this regard. Climate change is a major area of interest within the field of the sports industry (1). In recent years, there has been an increasing interest in climate change and the adaptations that the sports industry must make in order to not only seek and exploit opportunities, but also to recognize and respond to regulatory obligations (2). According to the authors, it is essential to integrate the natural environment into sports management strategies, interacting with other areas of sports management (e.g., marketing, psychology, and communication) to promote sustainable and informed practices, thus better preparing sports managers to face environmental challenges.

Despite growing interest, many organizations face difficulties in effectively integrating sustainable practices into their marketing strategies due to resource constraints, resistance to change, and a lack of specialized knowledge. Indeed, one of the most significant challenges for sports industry organizations is to define and implement their ethical, social, and environmental responsibilities; creating and seizing opportunities (e.g., brand differentiation and digital technologies), while avoiding potential pitfalls such as greenwashing or the use of sustainability for commercial purposes (3).

## 2 Overview of the papers in the special issue

In this special issue, four articles are outlined under the concept of the reality of climate change and the adaptations that sporting events need to face potential economic, social, and environmental consequences. We divided the presented papers according to four themes: i) a model for mega events; ii) the role of a large sports organization as a driver of sustainability; iii) a cross-sectional study of sustainability in various types of sports organizations; iv) the real impact of fan pressure on a sports organization.

### 2.1 A model for mega events

The importance of environmental sustainability in the sports sector is increasingly being discussed. The first article, by Cain, titled “*A figurational approach to environmental sustainability in the context of sport*” uses a sociological approach to propose a framework examining how policy development unfolds in the sports context, especially mega-events. Using the example of the Olympic Games, the four interrelated dimensions are presented and applied: lengthening chains of interdependence, established outsider power relations, internalization of social values, and unintended consequences. Since sport is a complex activity, composed of multiple relationships, the relevance given to the United Nations Sustainable Development Goals and their direct repercussions on the sports industry serves as a reminder of what is intended and what is possible. The process presented can facilitate a better understanding of the phenomenon in question. This study has important theoretical and practical applications in the context of climate change and the necessary adaptations that must be made by sports organizations, not only from a conceptual point of view, but also through the practical examples presented.

As Daddi et al. (4), point out in their work on how organization and management theories have been applied in climate change research, although the consistent application of management theories can enhance organizational strategies to address the climate crisis, “the relationship between business climate change strategies and other organizational theories still appears to be unexplored” (p. 456). However, it is a reality that sport can act as a driver of environmental change. In “The Green Waves of Environmental Sustainability in Sport,” McCullough et al. (5) highlight that sports organizations are increasing sustainable practices to reduce ecological impacts. This study also points out that sustainability in sport should be understood as a dynamic and collaborative process among institutions, athletes, and the public, emphasizing the role of fans and community engagement in promoting environmentally responsible behaviors.

### 2.2 The role of a large sports organization as a driver of sustainability

The second article conducts a policy review focused on the International Olympic Committee's (IOC) environmental sustainability agenda and sustainability initiatives (SI). Cain and Callan present “*Principles in Practice? A Policy Review of the IOC's Environmental Sustainability Agenda*.” As a global organization with the power to act as a driver of SI, the IOC has a significant impact on SI initiatives, both strategically and in terms of action, regarding desired objectives, policies, practices and principles. Through a study of various documents (e.g., policies, strategies, guidelines, reports, and conferences), the authors conclude that topics such as networks and knowledge transfer, leadership and influence, governance and accountability, and opportunities and obstacles emerge as important in the IOC's influence and performance. This study not only highlights opportunities to leverage stakeholders in SI better, but also points out flaws, inconsistencies, and some ambiguity in the work of this important global organization.

Daddi et al. (6), in their article “Exploring the Link Between Institutional Pressures and the Effectiveness of Environmental Management Systems: An Empirical Study,” investigate how institutional pressures influence the effectiveness of environmental management systems. The authors analyze organizations that have adopted environmental practices and identify regulatory, social, and market factors as key drivers. Compliance with external pressures enhances firms' credibility and environmental outcomes. For the authors, the interaction between institutional forces and internal management is essential to improving sustainable performance, especially given the “more positive influence of mimetic and normative pressure” (p. 1). In the sports industry, particularly in US college athletics departments, sustainability practices were examined by Casper et al. (7). By identifying levels of environmental awareness and actions taken to reduce impacts, the authors revealed that while some universities have clear policies, many still lack consistent strategies.

### 2.3 A cross-sectional study of sustainability in various types of sports organizations

The third article is titled “*The need to (climate) adapt: perceptions of German sports event planners on the imperative to address climate change*,” by Werner. Through a study of several sports organizations in Germany, the author aims to explore how sports events are impacted by climate change and identify measures for organizers to address these impacts. Based on a cohesive literature review and subsequent interviews with event planners and organizers of various sizes (local, national, and major events), the conclusion is clear and stark: climate change adaptation is not yet a primary focus in the German sports event context. This study shows, pragmatically, that the implementation of environmental measures by sports event organizers still has a long way to go.

Cayolla et al. (8), in their paper “If You Don't Know Me by Now – The Importance of Sustainability Initiative Awareness for Stakeholders of Professional Sports Organizations” explore the importance of awareness of sustainability initiatives (SIs) for stakeholders of professional sports organizations. They concluded that nearly one-third of stakeholders were unaware of the SIs implemented, indicating a critical need to improve communication about these initiatives. Trendafilova et al. (9) analyze the role of corporate social responsibility in promoting environmental sustainability in professional sports. On a more positive note, the authors highlight that leagues and clubs are increasingly adopting environmentally friendly practices as part of their image and social responsibility strategies. The study shows that these actions not only reduce environmental impacts but also strengthen reputation and fan engagement.

### 2.4 The real impact of fan pressure on a sports organization

The fourth article, a study of the most important stakeholder in sports (the fans), is conducted in a less studied region (South America, Colombia), in football, considered the most popular sport in the world (10). The article is titled “*Understanding fan pressures and its impact on football club sustainability: insights from the Colombian context*” by Hernández-Hernández et al. With a specific focus on the triple bottom line approach, the authors aimed to evaluate the impact of diverse fan types on the sustainability performance of Colombian football clubs. They conclude that, although evaluated positively, environmental engagement was the least relevant for fans, who prioritize social commitment as more important, followed by the promotion of women's football and the fight against racism. This study reinforces the importance of clubs' efforts to promote greater civic awareness beyond sustainable behavior during events, particularly through the triple bottom line dimension: social, environmental and economic.

In a similar vein, Cayolla et al. (11) investigated how fans perceive sustainability initiatives implemented by professional sports clubs and the subsequent impact on the triple bottom line perspective (TBL)—encompassing social, environmental, and economic dimensions. The study found that fans' perceptions of a club's pro-environmental initiatives positively influenced their social behaviors, such as increased environmental awareness and community engagement, as well as their daily environmental actions. Additionally, these perceptions enhanced fans' economic interactions with the club, including increased attendance and merchandise purchases. That underscores the potential for sports organizations to leverage environmental sustainability efforts not only to benefit the planet but also to strengthen their relationships with fans and enhance economic performance of the club. Previously, Casper et al. (12), also analyzed whether sustainability practices influence fan engagement and their connection to institutions. The results indicated that environmental efforts can improve organizational image and increase fan support. They concluded that investing in sustainability in sports not only contributes to the environment but also generates benefits in terms of public relations.

## 3 Research outlook and further research

In this special issue, the four articles presented provide relevant contributions to build sustainability models, suggest improvements in knowledge transfer, highlight the need to redirect the focus on sustainability regardless of the organization's size, and emphasize clubs' efforts to ensure fans have a greater understanding of the importance of sustainability. To avoid “new disciplinary silos” [(13), p. 1], simple actions such as annual reports, monitoring, controlling, and evaluating the initiatives carried out are crucial for better sustainable performance (14). Further exploration of foundational themes such as fan consumption, team-fan communications, and brand relationships are other suggestions for the future (3). Comparing additional audiences besides fans (e.g., young students), from different areas and different countries are also interesting topics of research (15, 16).

Technological advancements, such as artificial intelligence (AI), virtual reality (VR), augmented reality (AR) and extended reality (XR), are expected to evolve and play an even greater role in shaping customer/fan experiences, improving sports operations and supporting responsible initiatives. Future contributions should continuously explore the process of planning, creating, implementing and evaluating sustainability initiatives in sports using emerging technologies, guiding practitioners in developing effective and impactful responsible actions in sports.

In the field of sports, characterized by intense emotionality, the study of fans' emotions and how they can be encouraged through responsible initiatives from sports organizations are further avenues for coming developments. In addition, considering that many behaviors are collective (13), the importance of information and awareness campaigns about sustainability in everyday life becomes even more essential (17).
